# Implantes Mecânicos Valvares: Quais Seus Efeitos A Longo Prazo?

**DOI:** 10.36660/abc.20220875

**Published:** 2023-06-28

**Authors:** Eldys Myler Santos Marinho, Júlio Martinez Martinez Santos, Bruno da Silva Brito, Achilles de Souza Andrade, Johnnatas Mikael Lopes

**Affiliations:** 1 Universidade Federal do Vale do São Francisco Petrolina PE Brasil Universidade Federal do Vale do São Francisco (UNIVASF) – Educação Física, Petrolina, PE – Brasil; 2 Universidade Federal do Vale do São Francisco Paulo Afonso BA Brasil Universidade Federal do Vale do São Francisco (UNIVASF) – Medicina, Paulo Afonso, BA – Brasil; 3 Universidade Federal da Paraíba Programa de Pós-graduação em Neurociência João Pessoa PB Brasil Universidade Federal da Paraíba - Programa de Pós-graduação em Neurociência, João Pessoa, PB – Brasil; 4 Universidade Federal da Paraíba João Pessoa PB Brasil Universidade Federal da Paraíba – Medicina, João Pessoa, PB – Brasil

**Keywords:** Análise de Sobrevida, Implante de Prótese de Valva Cardíaca, Embolia, Trombose, Óbito

Procedimentos cirúrgicos para a substituição de estruturas corporais por meio de próteses talvez sejam as intervenções médicas que exigem grande perícia e monitoramento contínuo, prolongando a vida e a saúde de inúmeras pessoas. Este foi um tema da pesquisa intitulada “ *Aspectos Clínicos e de Sobrevida de Pacientes pós Implante de Valva Mecânica, com Ênfase em Trombose de Prótese Valvar* ” de Tagliari et al.^1^ publicado no volume 119, número 5 deste periódico.

Dentre as diversas contribuições que o estudo nos traz, há achados que precisam de mais esclarecimentos para que seja possível uma utilidade clínica e de saúde pública das intervenções de implante valvar mecânicas.

Iniciamos analisando os efeitos do tipo de prótese nos parâmetros ecocardiográficos. Os autores mostraram que ambos os grupos apresentaram aumento significativo do FEVE (%).^1^ Contudo, seria necessária uma comparação entre os grupos para melhor discernimento dos leitores, o que mostramos na tabela 1 , para uma avaliação da eficácia neste indicador. É possível afirmar que os grupos são semelhantes estatisticamente, como visto no manuscrito. Entretanto, o efeito dos transplantes de prótese na posição mitral e mitral-aórtico é pequeno no FEVE, pois o *d* de Cohen é menor que 0,50,^2^ enquanto o efeito da prótese aórtico é moderado. Isto faz surgir a pergunta: Qual a implicação dessa diferença de efeito clínico na conduta médica de seguimento, prognóstico e reabilitação física do paciente? As outras variáveis ecocardiográficas não permitem mais análise por ausência de dados estatísticos de razão crítica ou falta de comparabilidade.

**Tabela 1:** – Análise comparativa dos grupos de prótese valvar e seus efeitos clínicos

	Média antes	Média depois	Dp Antes	Dp Depois	Cohen d	IC95+	IC95-
Prótese posição Aórtica	54,1	62,6	14,7	12	0,64	54,13	62,57
Prótese posição Mitral	54,2	56,8	2,7	13,4	0,32	54,23	56,72
Prótese nas posições Mitro-aórtico	55,5	61,2	14,2	12,7	0,42	55,55	61,15

Dp: desvio-padrão; IC: intervalo de confiança.

Outro ponto que merece uma análise mais aprofundada é a análise de sobrevida.^3^ Este tipo de análise é aplicado para diversos eventos, mas numa abordagem descritiva de curvas de Kaplan Meyer o interesse sempre é o tempo em que 50% da amostra apresenta o desfecho ou algum período específico como um ano. No referido estudo,^1^ foram dois desfechos apresentados no método: óbito e trombose de prótese. Entretanto, na figura 3 do estudo em análise, não há como saber se se trata da sobrevida do desfecho óbito ou trombose. Subentende-se que seria o desfecho trombose devido a que o suplemento de gráficos se remete a este desfecho para outras variáveis independentes. Caso contrário, há incoerência na organização das evidências.

É fortemente interessante a apresentação das curvas de sobrevida para ambos os desfechos assim como explicitar o tempo mediano de sobrevida que auxilia na prática clínica e planejamento de ações no pós-cirúrgico. Destaca-se também que a curva de Kaplan Meyer apenas descreve o tempo de sobrevida e que é o teste de *Log Rank* que especifica a diferença entre o tempo de sobrevida nos grupos.^3^

Além da apresentação da análise bruta de sobrevida de ambos os desfechos e seus tempos de meia-vida, é também salutar a análise do óbito e da trombose valvar à luz da regressão de Cox para ajuste de efeitos pois somente foi apresentado para o desfecho óbito. Neste último caso, não ficou consistente se deveria se aplicar Cox de riscos proporcionais ou Cox tempo-dependente.^3^ Seria necessário apresentar a análise bruta para esse desfecho anteriormente.

Se o desfecho fosse a trombose, há elementos gráficos no suplemento para contraindicar o Cox de riscos proporcionais. Os gráficos de sobrevida sugerem riscos desproporcionais ao longo do tempo para os grupos das variáveis independentes analisadas. Adicionalmente, o *Hazard ratio* é uma medida de efeito que tem implicação sobre o tempo para o desfecho acontecer. Foi aplicada a interpretação do risco relativo que é a medida de efeito que incide sobre a probabilidade de o desfecho ocorrer e não sobre o tempo para a ocorrência do mesmo. Isto reflete sobre o achado ser útil no manejo tempo-dependente ou não.

Por fim, ficam dúvidas que ainda podem ser sanadas através de uma análise de sobrevida bruta e ajustada mais adequada como: as diferentes próteses têm efeito distintos no óbito e na trombose independente das características demográficas e clínicas da amostra? Quais variáveis clínicas têm efeito sobre os desfechos controlando a ação do implante? Como esses achados podem ser manejados durante a recuperação dos pacientes?
