# Supplementary material for: Implantes Mecânicos Valvares: Quais Seus Efeitos A Longo Prazo?
Source: Arq Bras Cardiol. 2023 Jun 28;120(6):e20220875. [Article in Portuguese] doi: 10.36660/abc.20220875 (PMC10364984; doi:10.36660/abc.20220875)
Supplement: Translation

# Translation: Mechanical Valve Implants: What Are Their Long-Term Effects?

**Authors:** Eldys Myler Santos Marinho, Júlio Martinez Martinez Santos, Bruno da Silva Brito, Achilles de Souza Andrade, Johnnatas Mikael Lopes

**Affiliations:** 1 Brazil Universidade Federal do Vale do São Francisco (UNIVASF) – Educação Física, Petrolina, PE – Brazil; 2 Brazil Universidade Federal do Vale do São Francisco (UNIVASF) – Medicina, Paulo Afonso, BA – Brazil; 3 Brazil Universidade Federal da Paraíba - Programa de Pós-graduação em Neurociência, João Pessoa, PB – Brazil; 4 Brazil Universidade Federal da Paraíba – Medicina, João Pessoa, PB – Brazil

**Keywords:** Survival Analysis, Heart Valve Prosthesis Implantation, Embolism, Thrombosis, Death

Surgical procedures for replacing body structures through prostheses are perhaps the medical interventions that require great expertise and continuous monitoring, prolonging the lives and health of countless people. This was a theme of the research entitled “ *Clinical Features and Survival Analysis of Patients after Mechanical Heart Valve Replacement, with an Emphasis on Prosthetic Valve Thrombosis* ” by Tagliari et al.^1^ published in volume 119, number 5 of this journal.

Among the various contributions the study brings us, some findings need further clarification so mechanical valve implant interventions can be useful clinically and in public health.

We started by analyzing the effects of the type of prosthesis on the echocardiographic parameters. The authors showed that both groups significantly increased LVEF (%).^1^ However, a comparison between the groups would be necessary for better discernment by the readers, which we show in Table 1 , for an evaluation of the effectiveness of this indicator. It is possible to state that the groups are statistically similar, as seen in the manuscript. However, the effect of prosthesis transplants in the mitral and mitral-aortic position is small on LVEF, as Cohen’s d is less than 0.50,^2^ whereas the effect of the aortic prosthesis is moderate. This raises the question: What is the implication of this difference in clinical effect on the medical management follow-up, prognosis, and physical rehabilitation of a patient? The other echocardiographic variables do not allow further analysis due to the absence of critical ratio statistical data or lack of comparability.

**Table 1:** – Comparative analysis of valve prosthesis groups and their clinical effects

	Media before	Media after	SD before	SD after	Cohen’s d	CI95+	CI95-
Prosthesis position Aortic	54.1	62.6	14.7	12	0.64	54.13	62.57
Prosthesis position Mitral	54.2	56.8	2.7	13.4	0.32	54.23	56.72
Prosthesis position Mitral and aortic	55.5	61.2	14.2	12.7	0.42	55.55	61.15

SD: standard deviation; CI: confidence interval.

Another point that deserves a more in-depth analysis is survival analysis.^3^ This type of analysis is applied to several events, but in a descriptive approach using Kaplan-Meyer curves, interest is always when 50% of the sample presents the outcome or some specific period, such as a year. In that study,^1^ two outcomes were presented in the method: death and prosthesis thrombosis. However, in figure 3 of the study under analysis, there is no way of knowing whether the survival is the death or thrombosis outcome. The outcome appears to be about thrombosis because the supplement graphics refer to it for other independent variables. Otherwise, there is inconsistency in the organization of evidence.

It is highly interesting to present the survival curves for both outcomes and explain the median survival time, which helps in clinical practice and planning of post-surgical actions. It is also noteworthy that the Kaplan-Meyer curve only describes the survival time and that the Log Rank test specifies the difference between the survival time in the groups.^3^

In addition to the presentation of the crude survival analysis of both outcomes and their half-life times, it is also important to analyze death and valve thrombosis in the light of Cox regression to adjust the effects since it was only presented for the outcome of death. In the latter case, it was not consistent whether to apply proportional hazards Cox or time-dependent Cox.^3^ It would be necessary to present the crude analysis for this outcome previously.

If the outcome was thrombosis, there are graphics in the supplement to contraindicate Cox proportional hazards. Survival graphs suggest disproportionate risks over time for the groups of independent variables analyzed. Additionally, the Hazard ratio is an effect measure that has implications on time for the outcome to occur. The relative risk interpretation was applied, which is the measure of the effect that focuses on the probability of the outcome occurring and not on time for its occurrence. This reflects on whether the finding is useful in time-dependent management or not.

Finally, some doubts can still be resolved through an adequate crude and adjusted survival analysis, such as: do the different prostheses have different effects on death and thrombosis, regardless of the demographic and clinical characteristics of the sample? What clinical variables affect outcomes controlling implant action? How can these findings be managed during patient recovery?
