# Supplementary material for: Implantes Mecânicos Valvares: Quais Seus Efeitos A Longo Prazo?
Source: Arq Bras Cardiol. 2023 Jun 28;120(6):e20220875. [Article in Portuguese] doi: 10.36660/abc.20220875 (PMC10364984; doi:10.36660/abc.20220875)
Supplement: Related texts

# Reply: Carta-resposta

**Authors:** Fábio Tagliari, Marcelo Goulart Correia, Guilherme Dalcol Amorim, Alexandre Siciliano Colafranceschi, João Manoel Pedroso, Luiz Fernando Rodrigues, Thaisa Rodrigues Tagliari, Clara Weksler, Cristiane Lamas

Agradecemos o interesse pelo nosso artigo “Aspectos Clínicos e de Sobrevida de Pacientes pós Implante de Valva Mecânica, com Ênfase em Trombose de Prótese Valvar”^1^ demonstrado pelos autores da carta. Em resposta aos questionamentos feitos, a respeito do aumento da fração de ejeção nos diferentes grupos de implantes protéticos, de fato nosso banco de dados carece de informações mais detalhadas para um estudo comparativo entre esses grupos. Seria necessária uma avaliação ecocardiográfica qualitativa de maior abrangência, com medidas diferentes para os diferentes tipos de doença valvar. Essa análise seria bastante complexa, dada a ampla apresentação da gravidade da cardiopatia orovalvar, seja univalvar, podendo a lesão ser pura ou mista, com predomínio ou não de insuficiência ou estenose valvar, e multivalvar, especialmente o comprometimento de mitral e aórtica, com diferentes apresentações hemodinâmicas e graus de gravidade,^2 - 4^ tempo de doença cardíaca orovalvar prolongado (observado mais nos pacientes reumáticos^5^ ), e pacientes submetidos a retroca valvar. Essas muitas variáveis limitam a comparação entre os pacientes. Contudo, podemos dizer que as lesões da válvula aórtica, sejam regurgitantes ou estenóticas, quando do início dos sintomas, costumam evoluir com uma melhora mais evidente da fração de ejeção após troca valvar, no acompanhamento tardio desses pacientes, já descritos na literatura clássica desde décadas passadas.^6^ Já as valvopatias mitrais apresentam-se com uma hemodinâmica diferente, seja por um ventrículo esquerdo preservado na lesão estenótica, ou por uma fração de ejeção pseudonormal, superestimada, na lesão regurgitante, mais evidente nos sintomáticos.^7 , 8^ Nas lesões mitrais, portanto, mesmo após troca valvar, não há aumento significativo da fração de ejeção no pós implante valvar protético em ambos tipos. Associa-se a isso diferentes graus de hipertensão arterial pulmonar, com ou sem disfunção ventricular direita e insuficiência tricúspide secundária, onde a intensidade das alterações estruturais miocárdicas conferem um prognóstico mais reservado a esses pacientes. Doenças valvares avançadas, mais perceptíveis em patologias da válvula mitral e combinadas, aórtica e valvar, apresentam prognósticos menos favoráveis em relação aos aórticos, com reabilitação mais prolongada, sendo os pacientes mais sintomáticos, de maneira geral, o que torna-se mais evidente quando coexiste disfunção ventricular direita já instalada.^6^ Desse modo, a indicação de cirurgia de troca valvar o mais precocemente possível, antes da degradação estrutural cardíaca, respeitando-se os riscos cirúrgicos inerentes, proporcionará os melhores desfechos clínicos de reabilitação e melhor prognóstico no seguimento médico ao paciente.^3 , 9^ A pressão sistólica de artéria pulmonar (PSAP) nos pareceu a mais interessante das variáveis ecocardiográficas para uma possível análise, contudo, por ser um banco de dados retrospectivo, tivemos muitos valores não aferidos (“missing”), como demonstrado na tabela 1 . De todo modo, ao analisarmos no grupo dos pacientes mitro-aórticos, as diferenças da PSAP 1, aferida no ecocardiograma pré cirurgia de troca valvar, com a PSAP2, aferida no último ecocardiograma disponível para o paciente, encontramos expressiva queda de valores, com significância estatística apenas para os mitroaórticos (média 40,5± 13,6 pré troca valvar vs. 32,6 ±10,2, p<0,001 pelo t de Student).

**Tabela 1:** – Resultados de pressão sistólica de artéria pulmonar em banco de dados de pacientes submetidos a troca valvar mecânica, INC 2011-2017

Categorias	Procedimento	N	Omisso	Média	Mediana	Desvio-padrão	Mínimo	Máximo
PSAP1	Aórtica	94	142	33,8	33	9,69	13	60
	Mitral	61	34	36,7	35	13,52	14	71
	Mitroaórtica	79	62	40,3	40	13,28	18	84
PSAP2	Aórtica	122	114	30,9	28	10,62	16	90
	Mitral	60	35	35,3	32,5	11,08	22	86
	Mitroaórtica	83	58	32,1	30	9,65	18	82

PSAP1: pressão sistólica de artéria pulmonar em ecocardiograma realizado antes da troca valvar; PSAP2: pressão sistólica de artéria pulmonar em ecocardiograma realizado após troca valvar no seguimento mais tardio do paciente

Em relação à análise de sobrevida, na figura 3 do artigo,^1^ a curva de sobrevida diz respeito a óbito por todas as causas. As curvas de Kaplan Meier para trombose de prótese estão mostradas nos gráficos suplementares 1 e 2, por gênero e por faixa etária.^1^ É importante ressaltar que tivemos apenas 5 pacientes com trombose, em 7 eventos. O evento trombose foi tardio, ocorrendo somente após o dia 2200. Não há massa de dados estatisticamente relevante para revelar diferença entre os grupos de substituição valvar mitral, aórtica ou mitro aórtica. A sobrevida mediana não foi atingida em nenhuma das três curvas.

Fizemos o modelo da regressão de Cox, tempo dependente, para a sobrevida/óbito, utilizando variáveis julgadas clinicamente relevantes. Encontramos que, controlando pela posição das próteses, o único fator que teve influência sobre o óbito ao longo do tempo foi a classe funcional, como vemos na tabela 2 . No gráfico suplementar 6 do artigo publicado já havíamos apresentado resultados semelhantes.^1^ Não encontramos, ao longo de nosso artigo, menção a risco relativo. Não é possível realizar uma regressão de Cox para o evento trombose dado o número pequeno de eventos.

**Tabela 2:** – Regressão de Cox para óbito de pacientes submetidos a troca valvar mecânica, INC 2011-2017

Desfechos tempo dependentes		Todos	HR (univariada)	HR (multivariada)
IRC	0	351 (96,7)	-	-
	1	12 (3,3)	5,00 (1,73-14,49, p=0,003)	2,44 (0,68-8,82, p=0,173)
CF	I	276 (76,0)	-	-
	II	77 (21,2)	5,20 (2,19-12,34, p<0,001)	**5,23 (2,11-12,94, p<0,001)**
	III	10 (2,8)	34,86 (13,37-90,89, p<0,001)	**39,94 (13,37-119,37, p<0,001)**
	IV	0 (0,0)	NA (NA-NA, p=NA)	NA (NA-NA, p=NA)
Procedimento	Aórtica	180 (49,6)	-	-
	Mitral	71 (19,6)	1,61 (0,56-4,64, p=0,382)	0,56 (0,14-2,17, p=0,398)
	Mitroaórtica	112 (30,9)	1,77 (0,79-3,96, p=0,162)	2,09 (0,68-6,39, p=0,198)
Etiologia Primária	Doença Reumática	244 (67,2)	-	-
	Bicúspide	51 (14,0)	0,47 (0,14-1,59, p=0,223)	1,00 (0,22-4,54, p=1,000)
	Calcificação/Degenerativa	53 (14,6)	0,34 (0,08-1,47, p=0,149)	0,56 (0,10-3,09, p=0,508)
	Indeterminada	14 (3,9)	1,43 (0,33-6,16, p=0,627)	1,02 (0,18-5,77, p=0,981)
Idade	Média (DP)	54,1 (10,8)	1,02 (0,99-1,06, p=0,197)	1,03 (0,99-1,08, p=0,122)

HR: hazards ration; IRC: insuficiência renal crônica; CF: classe funcional de acordo com a New York Heart Association (I-IV).

## References

[1] 1. Tagliari F , Correia MG , Amorim GD , Colafranceschi AS , Pedroso JM , Rodrigues Junior LF , Tagliari TR , Weksler C , Lamas C . Clinical Features and Survival Analysis of Patients after Mechanical Heart Valve Replacement, with an Emphasis on Prosthetic Valve Thrombosis . Arq Bras Cardiol . 2022 Nov ; 119 ( 5 ): 734 - 744 . English, Portuguese . doi: 10.36660/abc.20210544 .10.36660/abc.20210544PMC975022036453764

[2] 2. Tarasoutchi F , Montera MW , Grinberg M , et al . Diretriz Brasileira de Valvopatias-SBC2011/Diretriz interamericana de Valvopatias-SIAC 2011 . Arq. Bras. Cardiol . 97 ( suppl1 ).10.1590/s0066-782x201100200000122286365

[3] 3. Vahanian A , Beyersdorf F , Praz F , Milojevic M , Baldus S , Bauersachs J , et al ; ESC/EACTS Scientific Document Group . 2021 ESC/EACTS Guidelines for the management of valvular heart disease . Eur Heart J . 2022 Feb 12 ; 43 ( 7 ): 561 - 632 . doi: 10.1093/eurheartj/ehab395 .10.1093/eurheartj/ehab39534453165

[4] 4. Mantovani F , Fanti D , Tafciu E , et al . When Aortic Stenosis Is Not Alone: Epidemiology, Pathophysiology, Diagnosis and Management in Mixed and Combined Valvular Disease . Front Cardiovasc Med . 2021 Oct 15 ; 8 : 744497 .10.3389/fcvm.2021.744497PMC855403134722676

[5] 5. Barbosa PJB , Müller RE , Latado AL , et al . Diretrizes Brasileiras para Diagnóstico, Tratamento e Prevenção da Febre Reumática da Sociedade Brasileira de Cardiologia, da Sociedade Brasileira de Pediatria e da Sociedade Brasileira de Reumatologia . Arq Bras Cardiol . 2009 ; 93 ( 3 supl.4 ): 1 - 18 .20976376

[6] 6. Zipes , Peter Libby , Robert O. Bonow ; founding editor and online editor Eugene Braunwald . Braunwald’s Heart Disease: a Textbook of Cardiovascular Medicine. Philadelphia, PA : Elsevier/Saunders, 2015 .

[7] 7. Rosen SF , Borer JS , Hochreiter C , et al : Natural history of the asymptomatic patient with severe mitral regurgitation secondary to mitral valve prolapse and normal right and left ventricular performance . Am J Cardiol 74 : 374 , 1994 .10.1016/0002-9149(94)90406-58059701

[8] 8. Ling LH , Enriquez-Sarano M , Seward JB et al . Clinical outcome of mitral regurgitation due to flail leaflet . N Engl J Med 335 : 1417 , 1996 .10.1056/NEJM1996110733519028875918

[9] 9. Imasaka K , Tomita Y , Tanoue Y , Tominaga R et al . Early mitral valve surgery for chronic severe mitral regurgitation optimizes left ventricular performance and left ventricular mass regression . J Thorac Cardiovasc Surg . 2013 Jul ; 146 ( 1 ): 61 - 6 .10.1016/j.jtcvs.2012.05.05622717277

# Reply

**Authors:** Fábio Tagliari, Marcelo Goulart Correia, Guilherme Dalcol Amorim, Alexandre Siciliano Colafranceschi, João Manoel Pedroso, Luiz Fernando Rodrigues, Thaisa Rodrigues Tagliari, Clara Weksler, Cristiane Lamas

Thank you for your interest in our article “Clinical and Survival Aspects of Patients after Mechanical Valve Implantation, with Emphasis on Prosthetic Valve Thrombosis”^1^ demonstrated by the authors’ letter. In response to the questions raised regarding the increase in ejection fraction in the different groups of prosthetic implants, our database actually lacks more detailed information for a comparative study between these groups. A more comprehensive qualitative echocardiographic evaluation would be necessary, with different measures for the different types of valve disease. This analysis would be fairly complex, given the broad presentation of the severity of heart valve diseases, whether univalvular, with the lesion being pure or mixed, with or without predominance of valve insufficiency or stenosis, and multivalvular, especially mitral and aortic involvement, with different hemodynamic presentations and degrees of severity,^2 - 4^ prolonged time of heart valve diseases (more often seen in rheumatic patients^5^ ), and patients undergoing valve replacement. These many variables limit the comparison between patients. However, we can say that aortic valve lesions, whether regurgitant or stenotic, at the onset of symptoms usually evolve with a more evident improvement in the ejection fraction after valve replacement in the late follow-up of these patients, already described in the classic literature for decades. Mitral valve diseases present with different hemodynamics, either due to a preserved left ventricle in the stenotic lesion or an overestimated pseudonormal ejection fraction in the regurgitant lesion, which is more evident in symptomatic patients.^7 , 8^ In mitral lesions, therefore, even after valve replacement, there is no significant increase in ejection fraction after prosthetic valve implantation in both stenotic and regurgitant lesions. Associated with this, there may be different degrees of pulmonary arterial hypertension, with or without right ventricular dysfunction and secondary tricuspid insufficiency, where the intensity of myocardial structural alterations confer a more reserved prognosis to these patients. Advanced valve diseases, more noticeable in mitral valve pathologies and combined aortic and mitral valve, present less favorable prognoses when compared to aortic ones, with longer rehabilitation, with patients being more symptomatic, in general, which is more evident when patients already have right ventricular dysfunction.^6^ Thus, the indication of valve replacement surgery as early as possible, before cardiac structural degradation, respecting the inherent surgical risks, will provide the best clinical outcomes of rehabilitation and a better prognosis in the patient’s medical follow-up.^3 , 9^ Pulmonary artery systolic pressure (PASP) seemed to us the most interesting of the echocardiographic variables for a possible analysis; however, as it is a retrospective database, we had many values not measured (“missing”), as shown in Table 1 . In any case, when analyzing the group of mitral-aortic patients, the differences between PASP1, measured on the echocardiogram before valve replacement surgery, with PASP2, measured on the last echocardiogram available for the patient, showed a significant drop in values, with statistical significance only for patients with mitroaortic valve replacement (mean 40.5±13.6 pre-valve replacement vs. 32.6 ±10.2, p<0.001 by Student’s t)

**Table 1:** – Results of pulmonary artery systolic pressure in a database of patients undergoing mechanical valve replacement, INC 2011-2017

Categories	Procedure	No.	Missing	Average	Median	Standard deviation	Minimum	Maximum
PASP1	Aortic	94	142	33,8	33	9.69	13	60
	Mitral	61	34	36,7	35	13.52	14	71
	Mitroaortic	79	62	40,3	40	13.28	18	84
PASP2	Aortic	122	114	30,9	28	10.62	16	90
	Mitral	60	35	35,3	32,5	11.08	22	86
	Mitroaortic	83	58	32,1	30	9.65	18	82

PASP1: pulmonary artery systolic pressure on echocardiogram performed before valve replacement; PASP2: pulmonary artery systolic pressure on echocardiogram performed after valve replacement in the patient’s later follow-up.

Regarding the survival analysis, in Figure 3 of the article^1^ , the survival curve refers to death from all causes. The Kaplan Meier curves for prosthesis thrombosis are shown in supplementary graphs 1 and 2 by gender and age group.^1^ It is important to emphasize that we had only 5 patients with thrombosis in 7 events. The thrombosis event was late, occurring only after day 2200. There is no statistically relevant mass of data to reveal a difference between the mitral, aortic, or mitral aortic valve replacement groups. Median survival was not reached in any of the three curves.

We used a time-dependent Cox regression model for survival/death using variables judged to be clinically relevant. We found that controlling for the position of the prostheses, the only factor influencing death over time was the functional class, as shown in Table 2 . In supplementary graph 6 of the published article, we had already presented similar results.^1^ We did not find, throughout our article, mention of relative risk. It is not possible to perform a Cox regression for the thrombosis event due to the small number of events.

**Table 2:** – Cox regression for deaths of patients undergoing mechanical valve replacement, INC 2011-2017

Time-dependent outcomes		All	HR (univariate)	HR (multivariate)
CRF	0	351 (96.7)	-	-
	1	12 (3.3)	5.00 (1.73-14.49, p=0.003)	2.44 (0.68-8.82, p=0.173)
FC	I	276 (76.0)	-	-
	II	77 (21.2)	5.20 (2.19-12.34, p<0.001)	**5.23 (2.11-12.94, p<0.001)**
	III	10 (2.8)	34.86 (13.37-90.89, p<0.001)	**39.94 (13.37-119.37, p<0.001)**
	IV	0 (0.0)	NA (NA-NA, p=NA)	NA (NA-NA, p=NA)
Procedure	Aortic	180 (49.6)	-	-
	Mitral	71 (19.6)	1.61 (0.56-4.64, p=0.382)	0.56 (0.14-2.17, p=0.398)
	Mitroaortic	112 (30.9)	1.77 (0.79-3.96, p=0.162)	2.09 (0.68-6.39, p=0.198)
Primary Etiology	Rheumatic Disease	244 (67.2)	-	-
	Bicuspid	51 (14.0)	0.47 (0.14-1.59, p=0.223)	1.00 (0.22-4.54, p=1.000)
	Calcification/Degenerative	53 (14.6)	0.34 (0.08-1.47, p=0.149)	0.56 (0.10-3.09, p=0.508)
	Undetermined	14 (3.9)	1.43 (0.33-6.16, p=0.627)	1.02 (0.18-5.77, p=0.981)
Age	Average (SD)	54.1 (10.8)	1.02 (0.99-1.06, p=0.197)	1.03 (0.99-1.08, p=0.122)

HR: hazard ratio; CRF: chronic renal failure; FC: functional class according to the New York Heart Association (I-IV).
